# Mesothelioma of tunica vaginalis of "uncertain malignant potential" - an evolving concept: case report and review of the literature

**DOI:** 10.1186/1746-1596-6-78

**Published:** 2011-08-25

**Authors:** Kiril Trpkov, Richard Barr, Andrew Kulaga, Asli Yilmaz

**Affiliations:** 1 Department of Pathology and Laboratory Medicine, Calgary Laboratory Services and University of Calgary, Rockyview General Hospital, 7007 14 st, Calgary, T2V 1P9, Alberta, Canada; 2 Division of Urology, Southern Alberta Urology Institute and Alberta Health Services, Rockyview General Hospital, 7007 14 st, Calgary, T2V 1P9, Alberta, Canada

**Keywords:** mesothelioma, tunica vaginalis, well-differentiated papillary mesothelioma, malignant mesothelioma

## Abstract

Mesothelioma of tunica vaginalis is a rare neoplasm, typically demonstrating frankly malignant morphology and aggressive behavior. Rare cases of well-differentiated papillary mesotheliomas have also been reported, which, in contrast, demonstrate indolent behavior. There are, however, cases which do not fit into the well-differentiated or diffuse malignant mesothelioma categories and can be considered mesothelioma of tunica vaginalis of "uncertain malignant potential", which is an emerging diagnostic category. A 57-year-old man presented with a neoplasm in a hydrocele sac. The neoplasm was non-invasive, but showed focal complex and solid growth and it was difficult to categorize either as well-differentiated papillary mesotheliomas or malignant mesothelioma. After the initial limited resection, the patient underwent radical orchiectomy with hemiscrotectomy and is alive and without disease progression after 6 years. Documentation of these rare tumors will allow their distinction from true malignant mesotheliomas and will facilitate the development of specific treatment recommendations.

## Background

Mesothelioma of tunica vaginalis is a rare neoplasm comprising less than 1% of all mesotheliomas, with over 200 cases documented in the literature [1]. Diffuse malignant mesotheliomas of tunica vaginalis are highly aggressive tumors with rapid progression, resulting in local recurrence, regional and distant metastases and death. Over the years, a category of rare well-differentiated papillary mesotheliomas (WDPM) has emerged, which demonstrated indolent behavior, in stark contrast to diffuse malignant mesotheliomas. These neoplasms were reminiscent of the bland mesothelial tumors with similar morphology, which were first described in the peritoneal cavity of younger females [2]. Similar tumors were also described in the pleura, peritoneum and pericardium, and also in male patients [3,4]. By definition, WDPM of tunica vaginalis do not exhibit stromal invasion, which sometimes can be difficult to evaluate on microscopic examination [5]. It has been proposed recently that the designation WDPM be restricted only to WDPM of tunica vaginalis lacking any complex or adverse pathology, while the cases with more complex morphology that do not show overt signs of malignancy should be designated "mesothelioma of uncertain malignant potential" [6].

We present a case which highlights the difficulty of clearly categorizing a case either as WDPM or malignant mesothelioma, which reiterates the need of introducing a diagnostic category of mesothelioma of tunica vaginalis of "uncertain malignant potential". Currently, there are no specific treatment recommendations for WDPM or mesotheliomas of "uncertain malignant potential" and it is important that these uncommon tumors are well characterized and their long-term behavior documented, which will enable establishing evidence-based treatment guidelines.

## Case presentation

### Clinical history

A 57-year-old patient presented with asymptomatic hydrocele for 6 months prior to routine surgery. No prior history of local trauma or asbestos exposure was documented. Two small paratesticular nodular masses were found during the surgery, originating from the tunica vaginalis above the head of the epididymis, which measured 1.5 cm and 0.5 cm in diameter. The nodules were pedunculated and there was no gross evidence of invasion, resulting in limited local resection. Both masses had uniform yellow/tan cut surfaces on gross examination. Although a preliminary diagnosis of WDPM of tunica vaginalis was considered initially, the presence of some problematic morphologic features prompted an extramural consultation which acknowledged the difficulty in classifying the neoplasm. A close patient follow-up was recommended because of the inability to completely rule out the diagnosis of malignant mesothelioma. Therefore, a subsequent radical orchiectomy with hemiscrotectomy was performed five months after the initial resection, which included wide resection of the paratesticular and inguinal skin and soft tissue. The specimen showed no residual neoplasm and only three surface micronodules (all less than 1.5 mm) of reactive mesothelial hyperplasia were found. After a 6-year follow-up, the patient had no disease progression and no evidence of local recurrence or metastatic disease.

### Light microscopy

**Figure 1:**
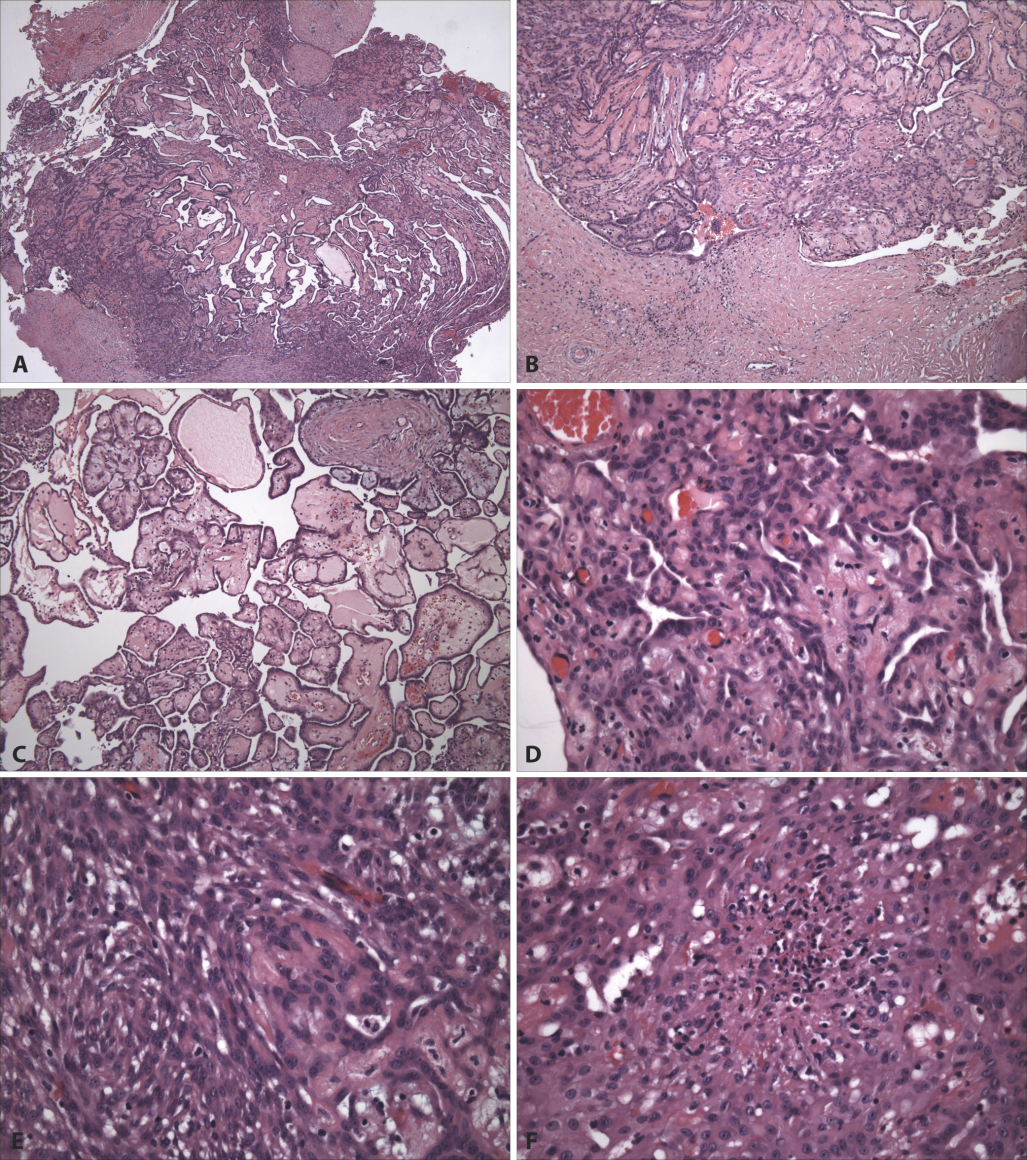
**Microscopic features of mesothelioma of uncertain malignant potential of tunica vaginalis**. **A**, Mesothelioma of tunica vaginalis. The smaller of the two resected tumors measuring 0.5 cm in diameter, is shown completely on low magnification (original magnification ×4). **B**, Both tumors were circumscribed and showed no evidence of invasion; the base of the stalk can be seen in the centre of the field (original magnification ×10). **C**, Both tumors were composed mostly of papillae with fibrovascular cores, lined by flat to cuboidal and focally columnar cells with bland nuclei. The fibrovascular cores were mostly paucicellular, but focally exhibited myxoid and hyalinized stroma (original magnification ×10). **D**, Focally, the neoplasm showed more complex growth with confluent cords and trabecullae (original magnification ×20). **E**, Smaller solid areas with spindle cells were also present (original magnification ×40). **F**, A single microfocus of coagulative necrosis was found in the larger tumor; this focus appeared in the vicinity of a stalk-like structure, raising the possibility of local torsion or infarct (original magnification ×40).

The specimen was fixed in neutral buffered formalin and was embedded routinely for histologic evaluation. Four-micrometer-thick sections were stained with hematoxylin and eosin. On light microscopy, both nodules resected initially were circumscribed and comprised mostly of variably sized papillary formations with fibrovascular cores, lined by flat to cuboidal and focally columnar cells with bland nuclei, showing no more than mild cytologic atypia (Figure 1A-C). The fibrovascular cores were mostly paucicellular and contained loose matrix, but focally exhibited myxoid and hyalinized stroma. Some papillae contained more cellular stroma with spindle cells. Parts of the papillary proliferations focally evolved into areas with more complex trabecular, syncitial and solid architecture (Figure 1D-E). The cells in these areas had uniform oval to spindle nuclei, exhibiting only mild atypia. No invasion was found into the adjacent stromal tissue, although only limited stroma was resected at the periphery. A single microfocus of coagulative necrosis was noted in the vicinity of a stalk-like structure the larger nodule (Figure 1F), raising the possibility of a local torsion or infarct. On complete sampling and multiple level examination, only one mitotic figure was found (<1 mitosis per 50 h.p.f.). No other adverse morphologic features were identified, including: more significant nuclear atypia, multinucleation, cell tufting, atypical mitoses, apoptosis, more extensive necrosis and no psammoma bodies were seen.

### Immunohistochemistry

On immunohistochemistry, the cells lining the papillae, as well as the cells in the areas of the more complex and solid growth, were uniformly immunoreactive for cytokeratin (pan -cytokeratin, AE1/AE3, CAM 5.2), calretinin, D2-40, WT-1 and vimentin, and were focally reactive for EMA and p53 (in approximately 10-20% of the tumor cells). Ki-67 was positive in less than 1% of the cells. Negative immunostains included: CEA, cytokeratin 5/6, Leu-M1 (CD15), Ber-Ep4 and MOC-31.

## Discussion

Mesothelioma of tunica vaginalis with dominant papillary architecture and focal complex morphology, but without other adverse findings, raises the dilemma whether it can still be considered as WDPM or whether it represents a predominantly papillary malignant mesothelioma. The neoplasm presented herein consisted of two small solitary tumors with mostly papillary architecture and bland cytology, but with no invasion into the surrounding tissue or infiltrative growth. The presence of some potentially adverse features, such as areas of complex and solid growth, a microfocus of coagulative necrosis and a mitotic figure, introduced the differential diagnosis of malignant mesothelioma with predominant papillary growth. The patient had an uneventful clinical course after a more radical surgery with no evidence of local disease recurrence or progression with distant metastases. It can be argued that a favorable outcome was achieved because of the radical treatment for a low-grade papillary malignant mesothelioma. Another option of resolving this diagnostic conundrum would be to consider this case a mesothelioma of "uncertain malignant potential", because it neither completely fits into the WDPM nor in the diffuse malignant mesothelioma category. This approach highlights the diagnostic uncertainty in these types of cases, particularly during the initial sign-out, when the invasive growth cannot be completely ruled out, particularly when dealing with a limited specimen or a limited excision.

WDPM of tunica vaginalis is considered "benign mesothelioma" in the World Health Organization fascicle on the tumors of the urinary system and male genital organs, primarily due to their indolent behavior [7]. A critical review of the literature, including reports with detailed microscopic descriptions and illustrations, revealed only 9 well-documented cases of WDPM, since the first description by Barbera and Rubino in 1957 [3,8-15]. WDPM were described in men between the ages of 18 to 70 years (median 56). All reported patients with WDPM had a good outcome with no evidence of disease recurrence or progression, although a relatively short follow-up of up to 3 years was provided for 7 out of 8 patients with available follow-up (median 21 months, mean 43 months; range, 12 to 120 months). Favorable outcome of WDPM of tunica vaginalis was achieved despite the variability of treatments, which included radical orchiectomy (5), limited local resection (4) or no surgery (1). The reported cases of WDPM arising in tunica vaginalis typically presented as solitary or, less often, limited number of superficial small nodules on the surface of a hydrocele sac, ranging from few mm to 3 cm in size, in contrast to the diffuse thickening or the multinodular neoplastic growth, typically seen in diffuse malignant mesotheliomas. On light microscopy, WDPM were typically composed of well-formed fibrovascular papillae, lined by a single row of flattened or cuboidal mesothelial cells, demonstrating bland cytology and low or absent mitotic activity (≤ 1 mitotic figure per10 h.p.f.). On immunohistochemistry, the cells uniformly marked as mesothelial and were reactive for cytokeratin, EMA, vimentin and calretinin.

The definition of WDPM has been somewhat controversial, but most experts currently restrict the term WDPM only to localized, solitary and exophytic tumors composed exclusively of delicate papillae lined by bland cuboidal cells [3,7,16]. Several of the previously reported WDPM of tunica vaginalis also included focal complex, tubulopapillary and solid morphology with confluent cords or genuine solid areas, but lacked overt signs of invasion or adverse morphology, similar to the case presented herein [2,8-13]. Others have also documented cases arising in the peritoneum and tunica vaginalis that contained more complex architectural features, making it difficult to clearly distinguish between WDPM and malignant mesothelioma [2,6,16,17]. Some of these cases were designated "papillary mesotheliomas with borderline features" or "localized mesotheliomas of low-grade malignancy", cautioning about their low malignant potential, despite the benign follow-up [2,3,11,16,17]. More recently, a term "mesothelioma of tunica vaginalis of uncertain malignant potential" was proposed for mesotheliomas with more complex morphology that do not fit the strict definition of benign WDPM or fulfill the criteria for diffuse malignant mesothelioma [6]. Currently, there are no evidence-based treatment recommendations for the tumors designated "WDPM or mesotheliomas of uncertain malignant potential" of tunica vaginalis, and it is important that these rare tumors are well documented with long-term follow-up.

The differential diagnosis of WDPM or mesotheliomas of uncertain malignant potential of tunica vaginalis includes primarily other mesothelial proliferations of tunica vaginalis, such as malignant mesothelioma, benign nodular mesothelial hyperplasia, adenomatoid tumor, and also some uncommon testicular or paratesticular neoplasms. However, it is crucial that they are distinguished from the malignant mesotheliomas, which typically present with multiple nodules of larger size, irregular nodules on the hydrocele surface or with diffuse and irregular thickening within a hydrocele. Less often, malignant mesotheliomas form solitary tumors or tumors with an exophytic, partially papillary growth, mimicking WDPM. Malignant mesotheliomas are often found to have hemorrhagic hydrocele fluid intraoperatively. On microscopy, they can be either epithelial, sarcomatoid or biphasic and they typically show frank invasion into the adjacent tissue including the testis, epididymis or the spermatic cord. Although the nuclei may demonstrate atypia and the nuclear size and shape may vary, many cases exhibit bland cytology. The mitotic figures are readily found and psammoma bodies may also be frequently seen. Local recurrences occur in 60% of the patients during the first two years after the initial treatment and in more than 90% of the patients within 5 years after the treatment [18]. In a comprehensive review of the literature of the malignant mesotheliomas, published in 1995, Plas et al found that the median patient survival was 23 months and for the patients with recurrent neoplasms only 14 months [18]. The cases associated with benign behavior demonstrated papillary growth, no more than mild cell atypia and absent or exceptionally rare mitotic figures [11]. The features of WDPM, mesothelioma of uncertain malignant potential and diffuse malignant mesothelioma arising in tunica vaginalis are summarized in Table 1.

**Table 1:** Comparison of the features of well differentiated papillary mesothelioma, mesothelioma of uncertain malignant potential and diffuse malignant mesothelioma arising in tunica vaginalis

	Well differentiated papillary mesothelioma	Mesothelioma of uncertain malignant potential	Diffuse malignant mesothelioma
**Clinical Presentation**	Hydrocele	Hydrocele (most often) or scrotal mass (rare)	Hydrocele or scrotal mass with hemorrhagic fluid
**Gross**	Single or limited number of small nodules (often pedunculated)	Single or limited number of small nodules (often pedunculated)	Diffuse thickening or multinodular growth (larger or irregular nodules)
**Microscopic Features**			
Papillary architecture	Yes (exclusive)	Yes (dominant)	Yes (focal or rarely dominant)
Complex morphology (cribriform, syncitial, solid architecture)	No	Focal	Yes
Stromal invasion	No	No	Yes
Cell atypia	No	No (or mild)	Yes
Mitotic figures	No (or exceptionally rare)	No (or exceptionally rare)	Yes
Atypical mitotic figures	No	No	Yes
Coagulative necrosis	No	No (or microfocal)	Yes
Sarcomatoid growth	No	No	Yes (if present, partial or dominant)
Psammoma bodies	No	No	Yes
**Clinical Behaviour**	Indolent	Indolent	Aggressive

The benign end of the spectrum of mesothelial proliferations is represented by nodular or reactive mesothelial hyperplasia which occurs most likely as a result of local irritation or injury, and is usually discovered incidentally in hernia sacs in children or in hydrocele sacs in adults. These are typically microscopic nodules or flat lesions on the surface of tunica vaginalis with bland appearance. They lack the distinctive papillary architecture seen in WDPM. Mesothelial reactions in hydroceles can sometimes exhibit diffuse and layered "zonal" distribution with entrapment of the mesothelial cells in a background of an organizing hematocele. These superficial extensions of mesothelial cells, glands and entrapped mesothelial cells are usually organized in parallel and linear arrays, which should not be confused with mesothelioma invasion [5,19].

Other rare paratesticular and testicular neoplasms should also be considered in the broader differential diagnosis of the mesothelial proliferations of tunica vaginalis and were previously reviewed comprehensively by Amin MB and Algaba and the collaborators [19,20]. These include the tumors of ovarian epithelial types (mostly serous papillary tumors of borderline malignancy), tumors of the collecting ducts and rete testis (mostly rete testis carcinoma) and papillary cystadenoma and adenocarcinoma of the epididymis. Lastly, when dealing with a paratesticular mass which may mimic a mesothelioma, possible metastatic deposits from common carcinomas, such as prostate, lung, colon, stomach, kidney and melanoma of the skin need to be ruled out. Metastatic tumors should particularly be considered if there is a clinical history of another primary or if the tumors are multifocal and show bilateral testicular involvement.

## Conclusions

We describe a mesothelial neoplasm which can be considered a mesothelioma of tunica vaginalis of uncertain malignant potential, an emerging diagnostic category, which can be applied to rare cases that do not fit into the categories of WDPM or diffuse malignant mesothelioma. The neoplasm was detected in a hydrocele and exhibited mostly papillary architecture with focal complex morphology, but did not show stromal invasion. Although no disease progression was documented on follow-up of 6 years after more extensive surgery, the appropriate treatment for these cases is uncertain, which highlights the necessity of their documentation with long term follow-up.

## Consent

Written informed consent was obtained from the patient for publication of this case report and any accompanying images. A copy of the written consent is available for review by the Editor-in-Chief of this journal.

## Abbreviations

WDPM: well-differentiated papillary mesothelioma

## Declaration of competing interests

The authors declare that they have no competing interests.

## Authors' contributions

KT conceived and designed the case report and drafted the manuscript. RB supplied clinical data and contributed to the revisions and editing of the manuscript. AK and AY participated in its design, contributed to the revisions and editing of the manuscript and provided images. All authors have read and approved the final manuscript.
